# Elective semaglutide prescription enabled waitlisting and transplantation of otherwise ineligible obese renal transplant candidates

**DOI:** 10.3389/frtra.2025.1623096

**Published:** 2025-10-29

**Authors:** Emilie Navaux, Caroline La, Sylvain Dufour, Vincent Huberty, Youssef Mourabit, Thomas Caes, Nikolaos Koliakos, Dimitri Mikhalski, Alain Le Moine, Concetta Catalano

**Affiliations:** ^1^ Department of Nephrology, Dialysis and Kidney Transplantation, HUB– Erasme, Université Libre de Bruxelles, Bruxelles, Belgium; ^2^ Department of Medical Gastroenterology, HUB - Erasme, Université Libre de Bruxelles, Bruxelles, Belgium; ^3^ Department of Gastroenterology, CHU-Charleroi Chimay, Université Libre de Bruxelles, Bruxelles, Belgium; ^4^ Department of Urology, HUB—Erasme, Université Libre de Bruxelles, Bruxelles, Belgium; ^5^ Department of Surgical Gastroenterology, HUB—Erasme, Université Libre de Bruxelles, Bruxelles, Belgium

**Keywords:** obesity, kidney transplantation, semaglutide, diabetes, dialysis

## Abstract

Although transplantation remains the treatment of choice for end-stage renal disease, patients suffering from severe obesity are too often unlisted for this reason. Pre-transplant bariatric surgery is not free of risk and the use of ‘Glucagon Like Peptide-1’analogues in these patients is limited. Our study aims to determine whether semaglutide administration enabled waitlisting and transplantation of otherwise ineligible obese renal transplant candidates. Between 01/01/2021 and 10/30/2023, patients rejected from renal transplantation because of obesity received pre-transplant subcutaneous semaglutide up to 1 mg/week. Of the 23 patients included, initial mean body weight, BMI and waist circumference were 102.9 Kg, 35.6 and 119.5 cm respectively. After a median of 12.2 months on semaglutide, these parameters decreased by 11.4 Kg (*p* ≤ 0.001), 3.9 points (*p* ≤ 0.001) and 9.6 cm (*p* ≤ 0.001) respectively. 56.5% of patients initially rejected for transplantation were listed within a median of 5.4 months, and 61.5% of them were transplanted. No major side effects were reported. In summary semaglutide administration enabled waitlisting and transplantation of otherwise ineligible obese renal transplant candidates. This treatment should be an integral part of the pre-transplant management of obesity.

## Introduction

Obesity, defined as a body mass index (BMI) ≥30, is a progressively increasing health concern. According to WHO data, numbers have more than doubled since 1990 and more than tripled since 1975 (1, 2). In patients with stage 4 and 5 chronic kidney disease (CKD) or on dialysis, obesity rate reaches 50% but, unlike the general population, patients with higher BMI exhibit better survival compared to those with lower BMI due to improved nutritional status and physical performance (3–5). Kidney transplantation is the preferred treatment for end stage renal disease (ESRD). It offers better survival rates (80%–90% at 5 years compared to 40% with dialysis) (6), improves quality of life (7), and lowers societal costs (8). As with advanced CKD patients, obesity increases among kidney transplant candidates (9) and hinders access to transplantation due to the higher incidence of postoperative complications (such as impaired wound healing, bacterial infections, delayed graft function and prolonged warm ischemia time) (2, 9, 10). However, the impact of obesity on patient and graft survival remains under debate (10).

Over the last few years, Glucagon-like peptide-1 receptor agonists (GLP-1 RAs), including semaglutide and liraglutide, have emerged as alternatives to surgery to achieve effective weight loss in patients living with obesity. Their main mechanism of action is to promote glucose-dependent insulin secretion in the pancreatic beta cells but they also slow gastric emptying and increase the feeling of satiety by acting on the hypothalamus through a central anorectic effect (11, 12). Initially reserved for diabetic patients, they were first studied in 2015 for their effect on body-weight (13).

Several studies have since demonstrated their efficacy in reducing body weight both in diabetic and non-diabetic patients with either low cardiovascular risk (14, 15) or with established cardiovascular disease (16). Data about efficacy and safety of semaglutide in kidney transplant candidates are limited to few case reports (17, 18). The aim of our study is to determine whether the elective prescription of GLP-1 RAs in a cohort of obese kidney transplant candidates results in sufficient weight loss to facilitate the access to transplantation.

## Materials and methods

The study was conducted between 01/01/2021 and 10/30/2023 at Hôpital Universitaire de Bruxelles and was approved by the Institutional Review Board (reference P2024/313).

Adult patients (between 18 and 75 years) with ESRD undergoing renal replacement therapy who had been deemed ineligible for renal transplantation due to overweight or obesity based on body mass index (BMI) were evaluated for inclusion in the study.

Only patients who had failed to achieve weight loss with non-surgical interventions within the preceding six months were prescribed with subcutaneous semaglutide after being evaluated by a multidisciplinary committee comprising medical and surgical team members, a psychologist, an endocrinologist, and a nutritionist.

Patients younger than 18 years or unwilling to participate in the study were excluded.

Patients’ demographic and clinical characteristics were retrospectively collected from the initiation of treatment and subsequently every three months until the end of follow-up. GLP-1RAs were prescribed in accordance with the manufacturer's recommendations, starting at 0.25 mg per week and titrated up to a maximum dose of 1 mg per week, based on patient tolerance. Semaglutide treatment was administered for a minimum duration of three months.

Obesity was defined according to World Health Organization (WHO) classification (1).

Body mass index was defined as body weight (in kilograms) divided by the square of body height (meters). Waist circumference was measured midway between the lowest ribs and the iliac crest and reported in centimeters (19).

At the end of follow-up, eligibility for renal transplantation was reassessed by both a nephrologist and a transplant surgeon. Data collection concluded at the time of wait-listing for patients who were listed and 12 months after inclusion for those who remained unlisted.

Data were recorded into a Microsoft Excel file according to European General Protection Regulation law and analyzed using SPSS statistical software.

Qualitative variables were expressed as percentages. Continuous parametric variables were expressed as means and standard deviations, while continuous non-parametric variables were expressed as medians and 25–75 percentiles. Paired-sample Student's *T*-test and paired-sample Wilcoxon–Mann–Whitney test were used to compare mean and median respectively.

## Results

Twenty-three patients have been included into the study. Cohort's characteristics at baseline and last follow up are summarized in Table 1. The mean age of the patients was 55 years, 61% of them were males and the median time on dialysis was 2 years. Fifteem patients (65.2%) had type II diabetes. At the beginning of the treatment, mean body weight, BMI and waist circumference were 102.9 Kg, 35.6 and 119.5 cm respectively. Median serum albumin was 41 g/L.

**Table 1:** Cohort's characteristics at baseline and last follow up.

Variables	At baseline	At last follow up	*p*
Age years—Mean (SD)	55 (13)		
Gender—*n*/*N* (%)			
Male	14/23 (61)		
Female	9/23 (39)		
BMI—Mean (SD)	35.6 (4)	31.7 (3.5)	<0.001
Points of BMI loss	NA	3.9 (2.4)	
Weight (Kg) Mean (SD)	102.9 (18.4)	91.5 (15.8)	<0.001
Points of weight loss		11.4 (7.2)	
Total weight loss (%)—Mean		10.8 (6.1)	
Overweight		5.6 (2.2)	
Grade I obesity		9.2 (5.5)	
Grade II obesity		13.1 (7.2)	
Grade III obesity		12.5 (6.1)	
Waist circumference (cm) Mean (SD)	119.5 (6.9)	109.1 (4.7)	<0.001
Waist circumference loss (cm)– Mean		9.6 (4)	
Diabetes– *n*/*N* (%)	15/23 (65.2)		
HbA1C (%)– Mean (SD)	6.7 (1.4)	6.0 (1.0)	0.019
LDL cholesterol mg/dl Mean (SD)	83.7 (31.8)	82.8 (33.9)	0.464
Serum albumin (g/L)—Median (P25-P75)	41 (37–43)	41 (38–43)	0,243
Serum pre-albumin (g/L)—Mean (SD)	0.31 (0.05)	0.33 (0.05)	0.017
GI symptoms—*n*/*N* (%)	3/23 (13)	5/23 (21.7)	

**Figure 1:**
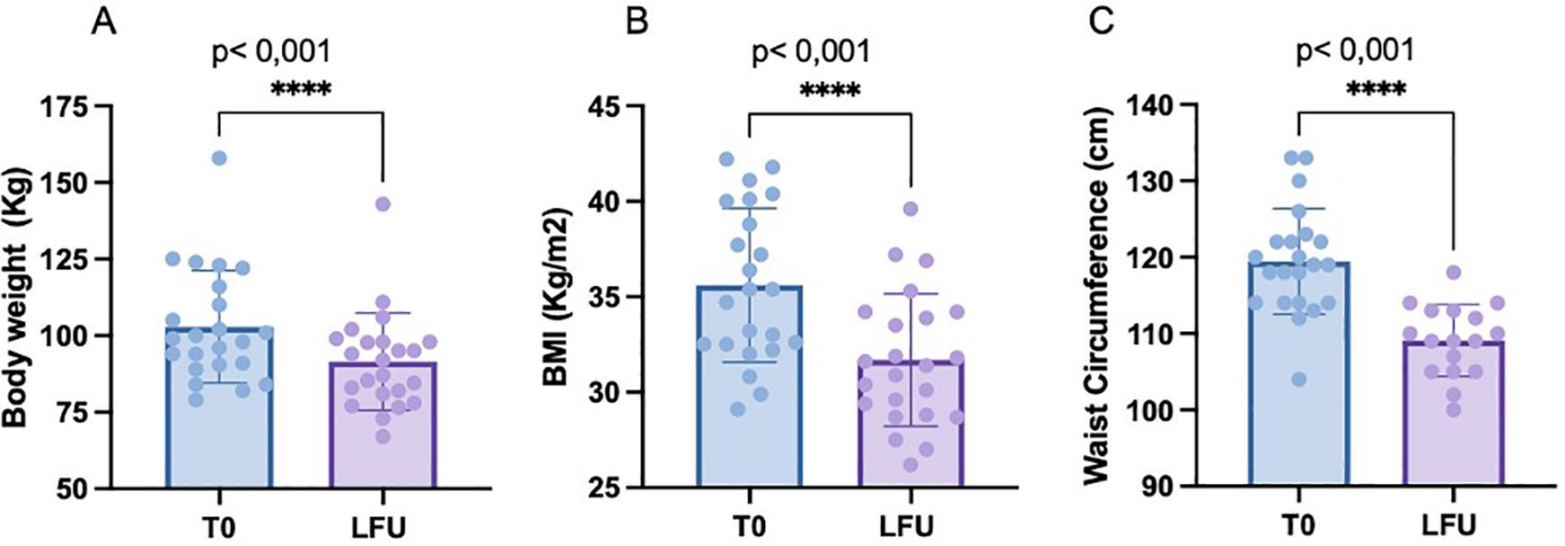
Illustrates the mean changes in weight **(A)**, BMI **(B)**, and waist circumference **(C)** between baseline (T0) and the last follow-up (LFU)”.

**Figure 2:**
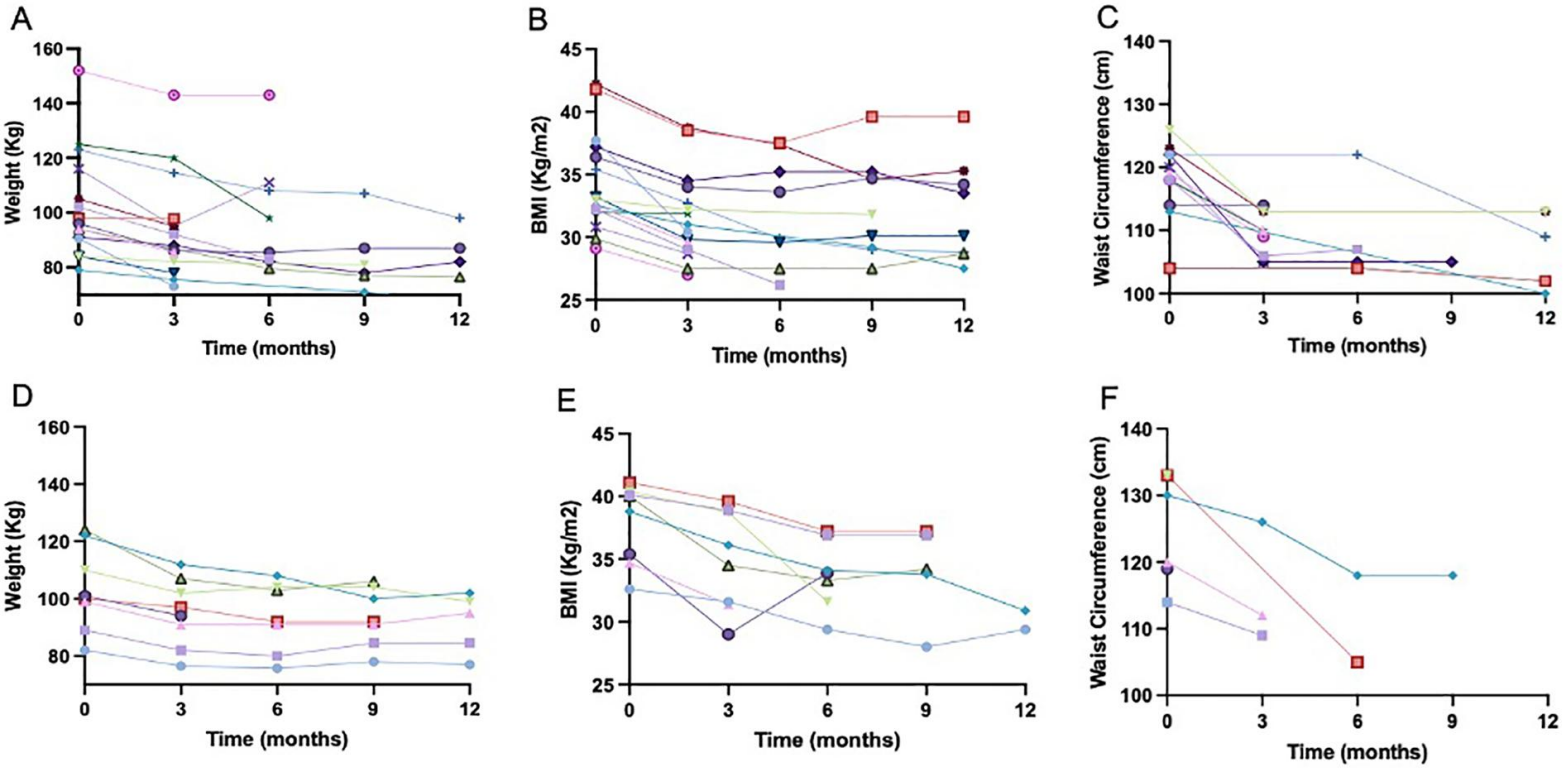
Illustrates the individual changes in weight, BMI and waist circumference in diabetic **(A–C)** and non-diabetic patients **(D–F)**. A distinct symbol–color combination was assigned to each patient.

After a median of 12.2 months of semaglutide (P25 5; P75 12.2), mean body weight, BMI and waist circumference decreased by 11.4 Kg (*p* ≤ 0.001), 3.9 points (*p* ≤ 0.001) and 9.6 cm (*p* ≤ 0.001) respectively (see Figure 1). Total weight loss (TWL%) was 10.8% in the whole cohort. TWL% according to the grade of obesity was 5.6; 9.2; 13.1 and 12.5% for overweight, grade 1-2-3 obesity, respectively. Mean weight loss according to diabetes status was 11.8 (6.7) and 8.9 Kg (4.5) in diabetic and non-diabetic patients, respectively. The individual changes in weight, BMI and waist circumference in diabetic and non-diabetic patients are illustrated in Figure 2.

Median serum albumin was was 41 g/L at baseline and remained unchanged at the last follow up (*p* = 0.24). No major side effects related to semaglutide were reported. Mild gastro-intestinal symptoms were reported by 11/23 patients (47.8%). Eight on eleven patients (73%) reported nausea, 4/11 (36.4%) presented vomiting, 3/11 (27.3%) reported gastro-intestinal reflux, 2/11 patients (18.2%) had diarrhea and 1 patient (9.1%) reported abdominal discomfort.

The majority of side effects were observed at the beginning of the treatment. In 6/11 patients (54.5%) symptoms disappeared after a dose reduction. In one patient, semaglutide had to be withdrawn at 4 months due to digestive intolerance. Five out of 23 patients (21.7%) still had gastrointestinal symptoms at the last follow-up, mostly represented by nausea. No evidence of a negative impact of semaglutide on residual renal function was found in the patients’ medical records.

Thirteen patients (56.5%) were activated on the transplant waiting list in a median time of 5.4 months and 61.5% of them (8/13) were further transplanted in a median time of 3.9 months. Among the 10 patients (43.5%) who remained unlisted, 9 of them (90%) were considered to have insufficient weight loss to be listed. In 1/9 patients (11.1%) Weight loss was not achieved because semaglutide had to be stopped early due to its unaffordable cost, despite a good initial response and tolerance. More than half of unlisted patients (55.6%) remained with class 3 obesity. Among unlisted patients, 1/10 (10%) developed severe vascular calcifications making him permanently ineligible for transplantation.

## Discussion

A healthy diet, regular physical activity, and modifications of eating behavior should always be encouraged to prevent obesity among kidney transplant candidates. However, lifestyle interventions alone, even with the support of a specialized multidisciplinary team, often fail to achieve significant weight loss in this population (20, 21). Gill et al (22). demonstrated that the risk of death decreases after transplantation regardless of donor type and BMI, compared to wait-listed patients, suggesting that despite the higher risk of postoperative complications, transplantation should still be promoted in obese candidates (23, 24). Moreover, patients with obesity should be promptly wait-listed because they may develop serious dialysis and caridiovascular-related complications, that could make them permanently ineligible for transplantation (20).

GLP-1 RAs have introduced a novel therapeutic approach for obesity management in the general population. Given their established efficacy and safety in high-risk patients, it is reasonable to hypothesize that they can be safely used in obese ESRD patients requiring kidney transplantation. Although used off-label, semaglutide demonstrated promising outcomes in our cohort, achieving the objective of wait-listing in more than half of the patients.

Despite authorization by the Food and Drug Administration (FDA) in 2021 and European Medical Association (EMA) in 2022, these medications face restrictive reimbursement criteria in most countries due to the drug shortage. For instance in Belgium, the drug is theoretically accessible to patients suffering from type 2 diabetes or to those suffering from morbid obesity (BMI > 35 kg/m^2^ or >30 kg/m^2^ plus at least one obesity-related comorbidity) (25). However, in practice, full reimbursement is limited to patients with uncontrolled diabetes (glycated hemoglobin ≥7.5%) who are already receiving at least one oral anti-diabetic medication, including metformin, for a minimum of three months (26). Patients who do not meet these criteria must cover the cost out of pocket, posing a significant financial barrier. This restrictive policy is particularly concerning given that semaglutide has been shown to reduce major adverse cardiovascular events by 20% in obese adults with established cardiovascular disease, even in the absence of type 2 diabetes (16). Consequently, limiting access to this medication may disproportionately affect ESRD patients and transplant candidates, who are at exceptionally high cardiovascular risk and mortality.

If we consider the principles described in the framework for fair-allocation of GLP-1 RAs in time of shortage reported by Persad et al (27), obese renal transplant candidates should be among those to benefit from GLP-1 RAs because they might have many expected advantages from the treatment in terms of access to transplantation, management of cardiovascular risk and reduction of mortality. In our study, semaglutide was well tolerated, with no serious adverse events reported at the maximum dose of 1 mg. This relatively low dose may have played a role in minimizing side effects. Importantly, no patients exhibited biochemical signs of malnutrition, further supporting the safety of semaglutide in the ESRD population. A similar use of GLP1-RAs in renal transplant candidates has already been described in two case series reported by Touzot and Wallace (17, 18).

Moreover, Segev et al. (28) showed that the probability of receiving a transplant decreased with increasing obesity, for patients with a BMI greater than 35. This can be explained by the fact that, more than BMI, waist circumference was a real limitation for surgeons, leading them to consider the patient ineligible for transplantation.

In fact, BMI may not be the best or even a good parameter for defining risk related to body composition, as it is poor at discriminating the ratio of fat to lean tissue within body weight. Abdominal adiposity represents an accumulation of fat around the viscera and is more strongly associated with insulin resistance, diabetes and dyslipidaemia than peripheral or subcutaneous fat. Compared to BMI, surrogate measures of abdominal adiposity and segmental fat distribution, such as waist circumference, are better correlated with all-cause and cardiovascular death in the general population (23) and in ESRD patients (29).

A more interesting finding in our study isthe statistically significant reduction in waist circumference (9.6 cm, *p* ≤ 0.001) after treatment with Semaglutide. A larger waist circumference before transplantation was associated with 2 fold post-transplant mortality in the study of Kovesdy et al. (23) and represented an independent risk factor for the development of new onset diabetes after transplantation (NODAT) as reported by Dedinska et al. (30).

This study has several limitations. The small sample size (*n* = 23) limits the generalizability of the findings and the relatively short follow-up period (median 12.2 months) does not allow for assessment of long-term metabolic and transplant outcomes.

Additionally, the mean BMI at inclusion was 35.6 kg/m^2^ in our cohort, whereas many transplant centers, particularly in the U.S., use a higher cutoff (≥40 kg/m^2^) for obesity-related restrictions.

The long-term durability of weight loss with GLP-1 RAs remains uncertain. Unlike bariatric surgery, which can sustain >25% weight loss after 10 years (31), discontinuation of semaglutide often leads to weight regain (14, 32). Due to our small cohort size, we could not determine whether weight regain, if it occurred, was related to treatment cessation or transplant-related factors. While this is an important consideration, our study's primary focus was rapid wait-listing rather than long-term weight maintenance. Other key unanswered questions include the optimal duration of GLP-1 RA therapy and whether continuing treatment post-transplant could protect graft function (33).

Despite the limitations, this study, provides informations about the short term effectiveness and safety of semaglutide in achieving wait-listing of renal transplant candidates living with obesity. Given the high cardiovascular morbidity and mortality in this population, obese transplant candidates should have equal access to and reimbursement for semaglutide, similar to diabetic patients.

Although the use of semaglutide in obese renal transplant candidates is becoming increasingly common in most transplant centers, the number of publications on the subject remains limited. To our best knowledge, this is the largest patients series reported on the subject and this study may be helpful to guide clinicians in charge of this kind of patients.

Further studies with larger cohorts and extended follow-up are necessary to better evaluate the long-term outcomes in this population.

In summary, semaglutide administration enabled waitlisting and transplantation of otherwise ineligible obese renal transplant candidates in our cohort. According to our results, GLP-1 RAs should be integrated into the stepwise pre-transplant management of obesity. This study highlights the need for a more equitable prescription of GLP-1 RAs for all patients at high cardiovascular risk, including those with ESRD, regardless of their diabetic status.

## Data Availability

The raw data supporting the conclusions of this article will be made available by the authors, without undue reservation.
